# Faces in shadows: silhouette light, underlight and toplight elicit increased early posterior negativity

**DOI:** 10.3389/fnins.2025.1553977

**Published:** 2025-03-10

**Authors:** Sampsa Huttunen

**Affiliations:** ^1^Faculty of Arts and the Department of Philosophy, History, and Art, University of Helsinki, Helsinki, Finland; ^2^Cognitive Brain Research Unit, Centre of Excellence in Music, Mind, Body and Brain, Department of Psychology and Logopedics, Medicum, Faculty of Medicine, University of Helsinki, Helsinki, Finland

**Keywords:** film lighting, character lighting, figure lighting, human face, subliminal emotions, electroencephalography (EEG), event-related potentials (ERP), early posterior negativity (EPN)

## Abstract

One key aspect of film lighting, and light in general, is its direction and how it illuminates people and other objects of attention. This research article presents the results of a pilot EEG experiment that studied the emotional responses of nine test subjects to photographs of an expressionless human face lit from varying directions. The aim of the study was to examine, how the direction of the main light source illuminating the face—the so-called ‘key light’ in filmmaking—would affect the test subjects’ subliminal-level emotional response before any conscious emotional processing takes place. EEG studies on how facial lighting affects the viewers’ subliminal emotions have not been reported in academic literature but, on the other hand, facial expressions and other emotion-eliciting visuals have been studied extensively. Based on a number of previous studies on subliminal emotions, the Early Posterior Negativity (EPN) measured in the occipito-parietal area of the scalp was chosen as the event-related potential (ERP) of interest, as it has been reported to reflect the subliminal processing of faces, facial expressions, and other visuals of evolutionary interest such as dangerous animals. Three light directions, (1) silhouette light that completely hides facial features, (2) underlight that comes from below the face and distorts those features, and (3) toplight that hides the eyes, were found to elicit a statistically more negative EPN than 45-degree light, a lighting style that reveals the whole face, gives the subject depth and separation from the background, and is therefore often used as the chosen key light direction in filmmaking and portrait photography, for example in the so-called three-point lighting technique. Contributing to cognitive film studies, these results indicate that the way a character’s face is lit affects the film experience as a whole already at the subliminal level of emotional processing.

## 1 Introduction

According to film scholars and filmmakers, light participates in telling a story in the same vein as any other element of the cinematic whole (Bordwell and Thompson, 2008; Monaco, 2009; Landau, 2014; Zettl, 2016; Brown, 2021, 2023). Among other things, these academics and practitioners point out that with light the filmmaker can emphasize certain story elements, reveal and hide things at particular moments during a scene, make characters, objects and places look appealing or appalling, and with all these aspects help shift the viewers’ feelings to the desired direction. Yet, how light was surrendered to the service of cinema relied on developments related to these physical features of light.

In scientific terms, the term “light” refers to the part of the electromagnetic spectrum that the human visual system can sense, and it consists approximately of all the radiation within the wavelengths from 380 to 750 nm. When innovators of the late 19th century learned to capture light on celluloid successively and with sufficient speed to create an illusion of movement, as these images were played back, the technology and art of cinematography were born.

The early processes of recording images needed significantly more light on the photographed target than the human visual system required to see it. Despite this early technical motivation for film lighting, already the first filmmakers begun to use light, not only to get a decent exposure on the very light-insensitive, or “slow,” film stocks of their time, but also, as artists, to express ideas on a visual level (Leitch, 2003; Keating, 2009, 2014). From early on, and especially after some technical innovations in indoor lighting such as spotlights (Bordwell et al., 1985), filmmakers have used light, among other things, to bring out desired elements and features, to guide the viewer’s attention among them, to create illusions of different times and places, and to establish a desired feeling or mood for the scene and the action being recorded (Leitch, 2003; Keating, 2009, 2014; Brown, 2023).

Today, film lighting as a part of the cinematographic process consists of several functions and features. This paper focuses on light’s directionality, the direction from which it falls on the people and other objects of attention. In nature, when light reaches an object, it usually has—apart from very cloudy or foggy situations—some level of directionality instead of being just ambient overall light. In cinematography this directionality is used, among other things, to enhance an object’s three-dimensionality by bringing out its shape and texture, to separate objects from their background, to help establish diegetic time and place (i.e., time and place existing within the story’s fictional world), and to emphasize psychological qualities of film characters and diegetic situations (see, e.g., Landau, 2014; Zettl, 2016; Brown, 2021, 2023).

When film lighting’s emotional functions are being discussed, the emotion-related notion of “mood” appears often in both theoretical and practical textbooks and articles about filmmaking and film lighting (Grodal, 2007; Pramaggiore and Wallis, 2008; Keating, 2009; Malkiewicz, 2012; Landau, 2014; Zettl, 2016; Brown, 2021). One of the first attempts (if not the very first) to give a more explicit meaning to this “mood” has been “Film Lighting and Mood,” a 2007 article by the Danish film scholar and professor Torben Grodal. In his article Grodal defines mood as the overall feeling the viewer gets when experiencing a film or a particular film scene. According to Grodal, this mood can be manipulated by the filmmaker through the use of light in a way that can either facilitate or restrict the viewer’s feeling of being able to cognitively engage with the diegetic environment presented in the film. In turn, the diegetic environment can be argued to provide the viewer “affordances” (Gibson, 1979) and, as proposed by Grodal (2007), these affordances, or the lack of them, can create emotional responses in the film viewers. These responses can be further enhanced through the viewer’s mirroring of the actions and emotions of the on-screen characters, as suggested by the Embodied Simulation theory (Gallese and Guerra, 2012).

Overall, the mood of a scene can be said to be the sum of all the affective elements the film viewers experience when they see and hear a particular diegetic setting, and of these, according to Grodal (2007), the angle of light and the composition of light within the frame are some of the most powerful tools for creating mood. Furthermore, the resulting affective whole consists, not only of the conscious feelings the spectator feels, but also of all the subliminal emotions that influence her perceptual–cognitive performance. These subliminal emotions, as they are related to the lighting of the face, are the object of this study.

In film lighting textbooks, courses, and web sites the single aspect receiving most attention is the lighting of the actor and especially the face. This is understandable, since just like in everyday face-to-face interaction, also in narrative films the actor’s face is often the center of our attention. In a previous study Huttunen (2022) studied film lighting’s conscious-level emotional effects. The study investigated whether the direction of light on the observed human face would affect the test subjects’ assessments of their own emotional reactions or how pleasant or unpleasant they rated the depicted face. The results of the study supported the hypothesis that any type of lighting that hides, obscures, or distorts facial information would result in more negative emotions and a lower pleasantness rating.

The hypotheses put forward by Huttunen (2022) are here surrendered to further examination. The aim is to clarify the possible role of character lighting in eliciting subliminal emotional responses in the viewer’s cortical areas by means of electroencephalography (EEG). These subliminal reactions have been shown to happen within a fraction of a second after the observed stimulus and before any conscious emotional processing can take place (Junghöfer et al., 2001; Schupp et al., 2006) and they are regarded as the earliest emotional reactions, which themselves can subsequently lead to conscious feelings (Damasio, 2001; Damasio and Carvalho, 2013).

At the heart of the present study is the hypothesis that certain types of lighting on a human face may elicit, not only conscious feelings, but also subliminal emotional responses. The hypothesis is based on the practical experience of professional cinematographers, the results of the previous experiment by Huttunen (2022), the studies of psychophysiological reactions to emotional faces (Schupp et al., 2004; Schindler and Bublatzky, 2020), and the fact that directional light always affects the visual features of the object it illuminates.

During our evolution we have been accustomed to light coming from above and seeing objects lit from above (Ramachandran, 1988; Morgenstern et al., 2011), and therefore, light coming from below can impede face recognition (Johnston et al., 1992; Favelle et al., 2017) or even make the face unrecognizable as a face (Palmer et al., 2022). As seeing and interpreting facial features is essential for our social communication (Zebrowitz, 1997; Fridlund and Russell, 2006) and in understanding and mirroring the other person’s thoughts and feelings (Dimberg and Öhman, 1996; Dimberg et al., 2000; Vuilleumier et al., 2001, 2002; Frith, 2009; Jack and Schyns, 2015), disrupting or hiding those features and the nonverbal information they provide may elicit emotions in us both on subliminal and conscious level.

## 2 Materials and methods

### 2.1 Stimuli

**Figure 1:**
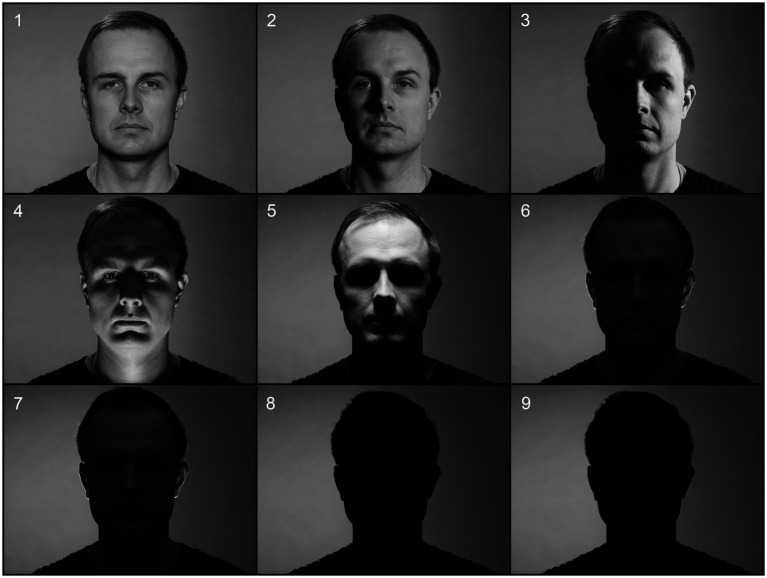
Experiment stimulus images of the nine lighting setups: (1) frontal light; (2) 45-degree light; (3) 90-degree light; (4) underlight; (5) toplight; (6) backlight; (7) backlight with eyelight; (8) silhouette light; (9) silhouette light with eyelight (Note that the eyelight in setups 7 and 9 might not be visible in a smaller-scale image).

The experimental stimuli consisted of nine black-and-white photographs of an expressionless male face (Figure 1) lit from different angles using a 40 × 40-cm LED (light-emitting diode) light source diffused with standard 1/2 white diffusion gel (Rosco) to make the light mimic natural daylight—a method used extensively in professional photography and cinematography. In all the setups the distance of the lighting unit from the subject’s head was 100 cm.

The distance of the camera from the subject’s eyes was also 100 cm. All test images were photographed using a Canon EOS 5D Mark II digital single-lens reflex (DSLR) camera and a Canon EF 50 mm f/1.8 STM prime lens. The camera sensor sensitivity was set to ISO 640, the aperture to f4, and the shutter speed to 1/160 of a second. All images were recorded using the camera’s own ‘neutral’ picture style (color and contrast settings) and saved as Canon RAW (.CR2) image files. For the frontal lighting in setup 1 (later: frontal light), the light was at eye level and right next to the camera. For the 45-degree side lighting in setup 2 (later: 45-degree light) and the 90-degree side lighting in setup 3 (later: 90-degree light), the light was at eye level 45 degrees and 90 degrees to the right from the subject, respectively. For the lighting from below in setup 4 (later: underlight) the light was on the ground level in front of the subject and for the lighting from above in setup 5 (later: toplight) above the subject. For the backlit setups 6 and 7 (later: backlight and backlight with eyelight respectively) the light was placed on to the ground level behind the subject and the diffusion gel was removed. The face in these setups was approximately four stops underexposed (i.e., receiving only 1/16 of the amount of light needed for technically correct exposure), which significantly obscured the facial features. For the silhouette lighting in setups 8 and 9 the light illuminating the subject was switched off altogether.

Setup 7 is a version of setup 6 with a small white spot added in each eye (later: eyelight), and setup 9 a similar version of setup 8. To keep the facial illumination exactly the same in both versions, the white spots of the eyelight were created in Photoshop (Adobe, Inc.). The placement of the spots was determined based on images photographed using an actual small light source creating the eyelight effect.

The reason for creating an eyelight version of the underexposed and silhouette image arises from the idea that a viewer can sense the presence of a person even in a very underexposed frame, if there is a glint of light shining from both eyes. The use of a separate eyelight is also often a preferred technique in film lighting, if none of the other lights reflect from the subject’s eyes – unless the filmmaker deliberately wants to imply that the character is not alive or is in some way evil (Thompson and Bowen, 2009) or wants to give the scene a menacing or mysterious mood (Lowell, 1992). The effect of eyelight on viewer attention and emotion discernment has been studied, for example, by Swenberg et al. (2022), whose results provide support for the idea that eyelight can enhance emotional communication in film and stills photography.

All images were taken in a photography studio in front of an evenly lit green chroma screen. Since study indicates that color may affect the way we interpret facial expressions (Young et al., 2013) and the same stimulus images were used in the previous experiment studying viewer’s conscious reactions (Huttunen, 2022), the color information of the photographs originally shot in color was removed. With regards to subliminal responses, study also indicates that there should be no difference in the EPN between color or grayscale images (Junghöfer et al., 2001).

The lightness level of the background was kept close to middle gray (18% reflectance), although some light from the key light was allowed to fall on the background to keep the look of the setting natural, meaning that light from a specific direction was falling on both the subject and the background. In setups 1 to 4, a small LED light was also placed above the subject to create a touch of light on the hair that would not interfere with the key light.

### 2.2 Participants

The test subjects consisted of nine people (4 female, 5 male) aged 20–80 years (mean age = 46.45, SD = 15.69). Eight of the test subjects were right-handed and one was left-handed (self-reported). All participants had normal or corrected-to-normal visual acuity (self-reported), had no known medical conditions, which might have affected the results, and provided informed consent to participate in the study.

### 2.3 The ERP of interest: early posterior negativity

The experimental setup of this study involves the measurement of the event-related potentials (ERPs) in the spontaneous electrical activity of the brain evoked by the stimuli and recorded as electrogram with electroencephalography (EEG). Earlier EEG studies of emotional content have indicated that the ERP components signaling early emotional processing of visual stimuli are P1 (also known as P100), N170, EPN, P3 (also known as P300), and the late positive potential (LPP) measured at the locations over and near the area of visual cortex in the occipital, parietal, and temporal lobe (Hajcak et al., 2011; Schindler and Bublatzky, 2020). Based on these studies, the early posterior negativity (EPN) was chosen as the ERP of interest, as it has been found to reflect early visual processing of emotional targets, such as dangerous animals (Van Strien et al., 2009, 2014; Langeslag and Van Strien, 2018) and human expressions of fear and anger (Schupp et al., 2004; Schindler and Bublatzky, 2020).

The EPN is a relative shift towards more negative values in scalp positivity. It is recorded over temporo-occipital or parieto-occipital sites and is believed to originate from the visual cortex and reflect increased activity of early visual processing (Schupp et al., 2003b, 2004). Numerous studies also indicate that the enhancement in the EPN is more pronounced for stimuli with high levels of emotional arousal than for neutral stimuli (Schupp and Kirmse, 2021), which is considered to reflect the involvement of higher attention and more detailed sensory processing (Junghöfer et al., 2001). Also, unlike some other event-related measurements of emotional reactions, such as skin conductance responses (SCR), the EPN is not modulated by habituation to stimuli (Schupp et al., 2006).

In previous studies the EPN has been linked to emotional processing in several different areas, such as, emotional scenes (Schupp et al., 2006), dangerous animals, such as snakes and spiders (Van Strien et al., 2009, 2014; Langeslag and Van Strien, 2018), erotic stimuli and mutilations (Schupp et al., 2003a; Schupp and Kirmse, 2021), facial expressions (Schupp et al., 2004; Schindler and Bublatzky, 2020), emotional words (Palazova et al., 2011; Kissler et al., 2007), emotional auditory stimuli (Mittermeier et al., 2011; Jaspers-Fayer et al., 2012), and even emotional hand gestures (Flaisch et al., 2009). Earlier EEG studies of emotion have also observed EPN in different time windows within an area spanning from 132 ms (Jaspers-Fayer et al., 2012) up to 600 ms after stimulus (Rellecke et al., 2012), although typically the EPN has begun approximately 150 ms after stimulus and reached its maximum negativity between 200 and 300 ms after stimulus (Junghöfer et al., 2001; Schupp et al., 2006; Hajcak et al., 2011).

### 2.4 Hypothesis

Based on the findings of the previous study of conscious emotional reactions (Huttunen, 2022) and a preliminary experiment, in which the skin conductance response (SCR) of six test subjects were measured while watching the same stimulus images (Figure 1), it was hypothesized that lighting setups that would elicit a pronounced negativity in the EPN would be underlight (setup 4) and silhouette light (setup 8).

It was also hypothesized that the emotionally most “neutral” and, therefore, least negativity-eliciting lighting setup would be 45-degree light (setup 2), since its light direction does not hide, obscure, or distort any facial features, creates pleasant-looking volume and three-dimensionality for the face, separates the subject from the background, and is, for these reasons, also used extensively as a typical key light direction in portrait photography and cinematography (Brown, 2023).

### 2.5 Measurements

The EEG measures were taken from 32 scalp electrodes based on the 10–10 system with ground electrode at Fpz using a BrainVision Recorder version 1.25.0201 (Brain Products GmbH), Easycap Standard 128 channel caps (Easycap GmbH), actiCAP snap active electrodes (Brainvision, LLC) and an actiCHamp Plus amplifier (Brain Products GmbH).

**Figure 2:**
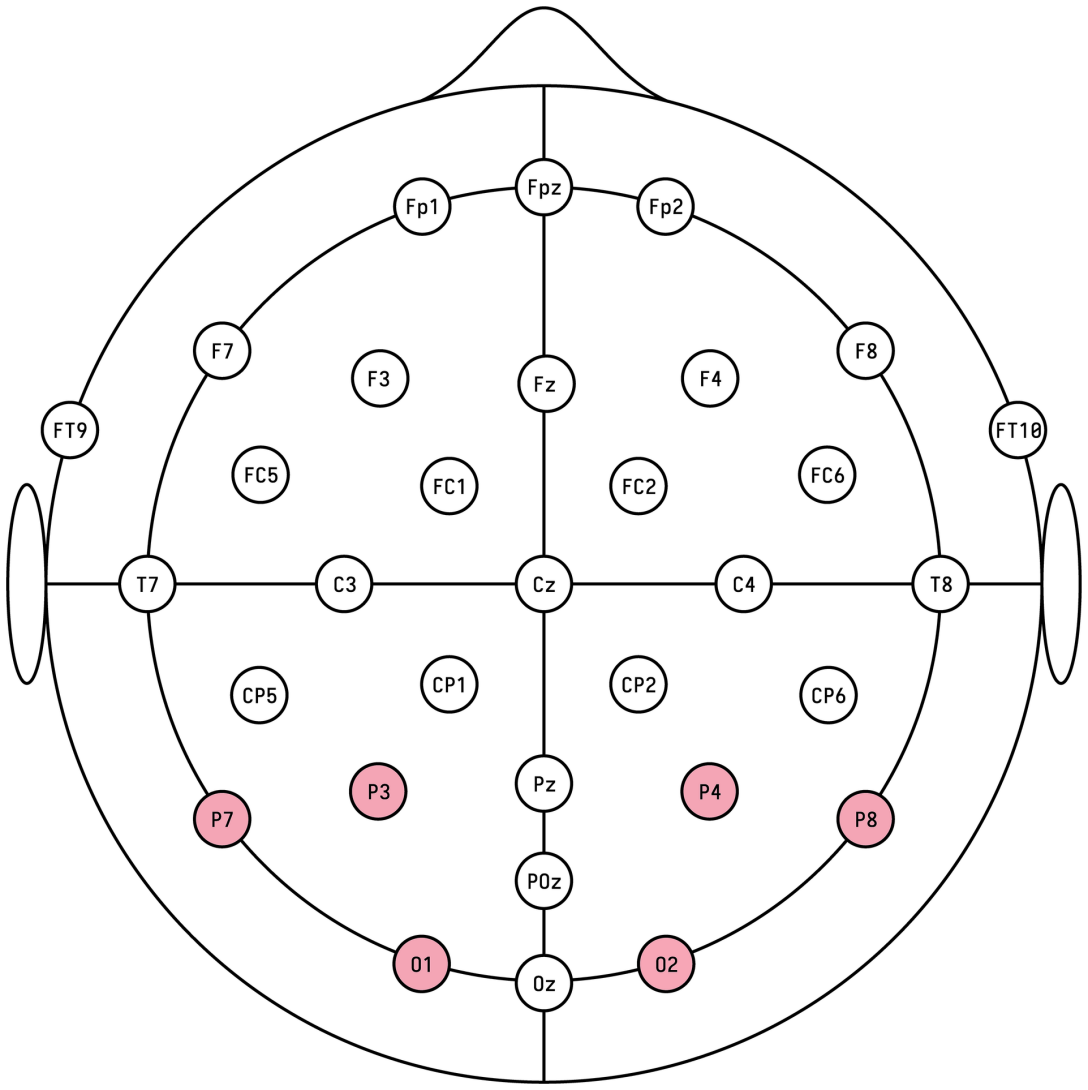
The occipito-parietal recording sites O1, O2, P3, P4, P7, and P8 formed the ROI in the experimental setup.

The region of interest (ROI) for the EPN was defined as the average activity of occipital recording sites O1 and O2 and parietal sites P3, P4, P7, and P8, as used in a previous study by Hajcak and Dennis (2009) (see Figure 2).

### 2.6 Experimental setup

**Figure 3:**
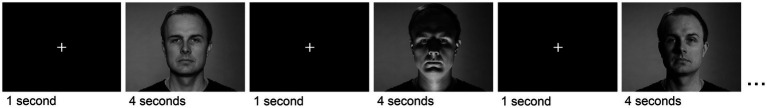
The stimulus photographs were presented in a randomized order, 4 s each, intercepted by 1 s of center cross.

The images were shown to the test subjects in a Faraday cage at the Cognitive Brain Research Unit (CBRU) lab of the University of Helsinki. Each stimulus photo was shown 20 times in a random order in a cycle containing 4 s of stimulus and 1 s of center cross (see Figure 2). Although an ERP response happens in a fraction of a second after the stimulus, each photo was presented for 4 s to make the test feel meaningful to the test subjects also at a conscious level (see Figure 3).

The stimulus images were presented on the laboratory microcomputer using Presentation software version 22.1 build 04.30.21 (Neurobehavioral Systems, Inc.) to control the presentation and timing of all stimuli. Each video image occupied 44.8 cm × 33.7 cm (resolution 1,440 × 1,080 pixels) on a 27-inch (69 cm) monitor (60 Hz refresh rate) that was visually calibrated for the right brightness and contrast settings. At a viewing distance of 80 cm, each picture occupied approximately 31 degrees of visual angle horizontally and 23 degrees vertically. The participants were instructed to sit still during the experiment and to look only at the center cross or the image presented on the computer screen. The center cross was positioned so that it appeared in the area between the eyes of the face depicted in the stimulus images. The experiment was monitored through a small web camera and a microphone, and the subjects were instructed to abort the experiment, if they felt any discomfort.

### 2.7 Signal treatment

All bioelectric signals were recorded using BrainVision Recorder version 1.25.0201 (Brain Products GmbH), and the EEG was sampled at 500 Hz. The off-line analysis of the data was performed using CBRU Plugin (version 2.1.6b), an in-house MATLAB-based analysis software using MATLAB software version R2023b, update 3 (The Mathworks, Inc.) and EEGLAB toolbox version 2023.0 (Delorme and Makeig, 2004).

**Figure 4:**
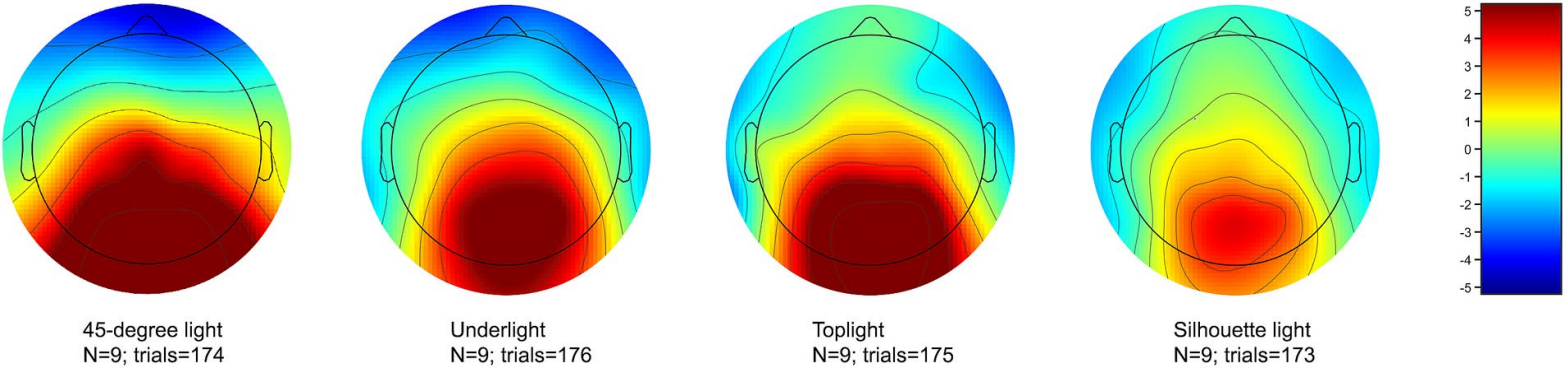
The mean voltage of all channels during the 150–300 ms analysis window of the EPN for 45-degree light (setup 2), underlight (setup 4), toplight (setup 5), and silhouette light (setup 8). The pronounced EPN can be seen as less positive values in the occipito-parietal areas of the scalp at the back of the head.

**Figure 5:**
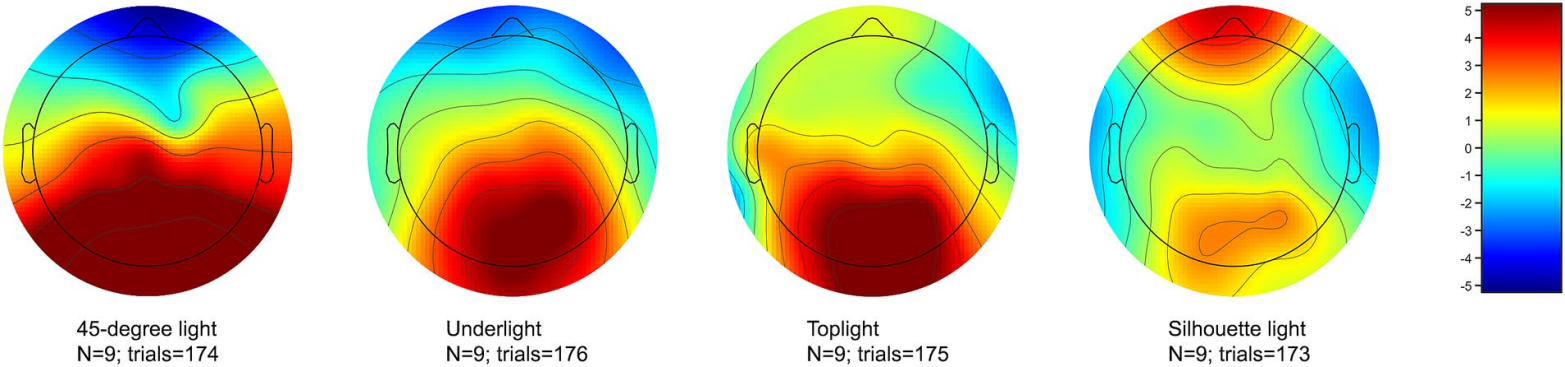
The mean voltage of all channels during the 200–600 ms analysis window of the EPN for 45-degree light (setup 2), underlight (setup 4), toplight (setup 5), and silhouette light (setup 8). The pronounced EPN can be seen as less positive values in the occipito-parietal areas of the scalp at the back of the head. The visible frontal positivity is due to uncleaned ocular artifacts affecting this area outside the ROI of the study.

The visual inspection of the signals showed that one participant had a noisy signal on the frontal and fronto-temporal channels F3 and FT10 and the other participant on the temporal channels T7 and T8, both probably due to insufficient contact between the electrode and the scalp. The data for these channels were interpolated from the neighboring channels using EEGLAB’s spherical interpolation method. On the other hand, the data were not separately cleaned for ocular artifacts, which mainly affect the frontal areas, and can be seen as frontal activity in the voltage maps of Figures 4, 5. No data from the frontal or temporal channels were used in statistical analyses, since these channels were outside the ROI, and used only in assembling the scalp voltage maps in Figures 4, 5.

All data were band-pass filtered with cutoffs of 0.1 Hz and 40 Hz, and the EEG was segmented into epochs for each trial, beginning 100 ms before each stimulus onset and continuing for 600 ms after stimulus onset. In each epoch, the pre-stimulus baseline was removed, and epochs with a baseline-to-peak amplitude difference larger than ±100 microvolts (μV) on any channel within the ROI were omitted from averaging. The epochs were averaged for each stimulus type within a participant, and further averaged across the channels selected for the ROI resulting in a time series representing a participant’s brain activity on the channel subset in each stimulus type.

### 2.8 Statistical tests

The ERPs were statistically evaluated using SPSS Version 28.0.0.0 (190) (IBM Corp., New York, NY). As the visual inspection of the individual data distribution revealed that not all data was normally distributed, nonparametric statistical tests were used in the statistical analysis.

First, the Friedman test was used to detect statistical differences in the mean negativity across all lighting setups within the time span of 150–300 ms. After that the paired nonparametric Wilcoxon signed-ranks test (later: Wilcoxon test) was used to compare lighting setups pairwise.

After the statistical tests of the 150–300-ms time span, the ERP signals were visually evaluated, which revealed visible differences also later in the mean negativity, approximately within the area of 200 to 600 ms post stimulus. Once again, the Friedman test was first applied also to this area of the signal followed by the pairwise Wilcoxon test.

## 3 Results

The performed Friedman test showed a statistically significant difference between the mean amplitudes of the nine lighting setups within the predetermined area of 150 to 300 ms after the stimulus onset [*χ*^2^(2) = 16.119, *p* = 0.041]. Then, the subsequent Wilcoxon test showed that the mean EPN elicited by underlight (setup 4) and silhouette light (setup 8) was statistically significantly more negative than that of 45-degree light (setup 2) (*Z* = −2.073, *p* = 0.038 and *Z* = −2.192, *p* = 0.028) indicating that these lighting setups elicited a stronger early emotional response than the more typical facial lighting from a 45-degree angle. Other lighting setups failed to reach statistical significance, interestingly also the eyelight version of silhouette light (setup 9)—a silhouette face with two small white dots in place of eyes. The test statistics of all pairwise comparisons against 45-degree light are presented in Table 1.

**Table 1:** Statistical comparisons of the signal means of 150–300 ms after stimulus using the Wilcoxon test; other lighting setups against 45-degree light.

Test statistics for 150–300 ms^a^								
	Frontal light vs 45° light	90° light vs 45° light	Underlight vs 45° light	Toplight vs 45° light	Backlight vs 45° light	Backlight + eyelight vs 45° light	Silhouette vs 45° light	Silhouette + eyelight vs 45° light
*Z*	−0.059^b^	−0.178^c^	-2.073^b^	−1.481^b^	−0.652^b^	−0.652^b^	−2.192^b^	−1.599^b^
Asymp. sig. (2-tailed)	0.953	0.859	0.038	0.139	0.515	0.515	0.028	0.110

a Wilcoxon signed ranks test.

b Based on positive ranks.

c Based on negative ranks.

*Z* scores are presented on the first row and two-tailed asymptotic significances (*p*-values) on the second row. The statistically significant differences in the EPN of underlight (setup 4) and silhouette light (setup 8) compared to 45-degree light (setup 2) are highlighted in yellow and suggest that these lighting styles elicit a more pronounced negativity and therefore a stronger emotional response than the more typical facial lighting of 45-degree light.

**Figure 6:**
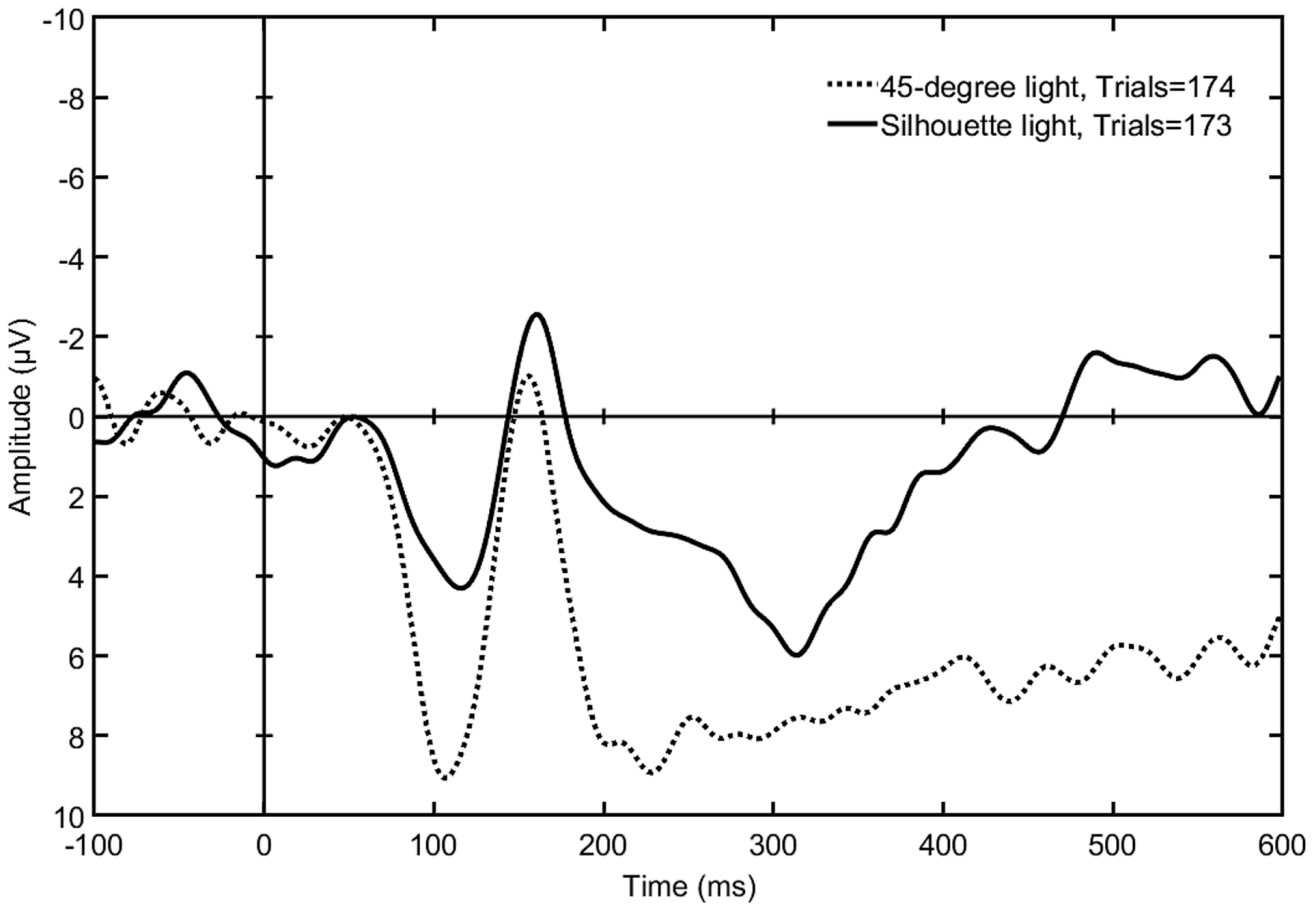
The mean signal amplitudes elicited by silhouette light (setup 8) and 45-degree light (setup 2) (*N* = 9). The graph shows a clear difference in the mean negativity between these lighting styles within 150–600 ms after stimulus, which indicates that a lighting that leaves the face in complete darkness elicits a stronger early emotional reaction than a lighting that reveals the whole face.

**Figure 7:**
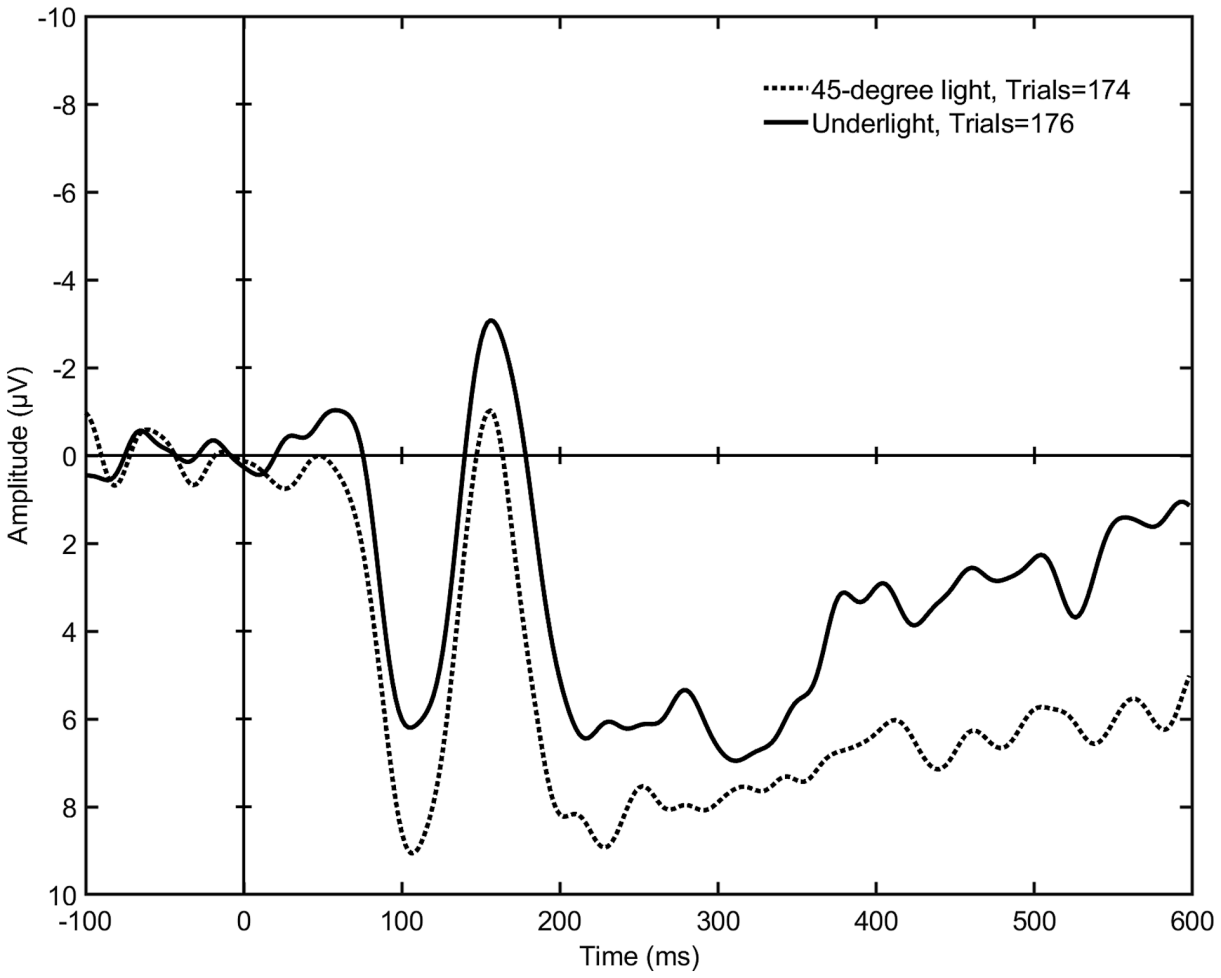
The mean signal amplitudes elicited by underlight (setup 4) and 45-degree light (setup 2) (*N* = 9). The graph shows a visible difference in the mean negativity within both 150–300 and 200–600 ms after stimulus, although the latter failed to reach a statistical significance. This indicates that a lighting that comes from below the face distorting the facial features elicits a stronger early emotional reaction than a lighting that reveals the whole face.

**Figure 8:**
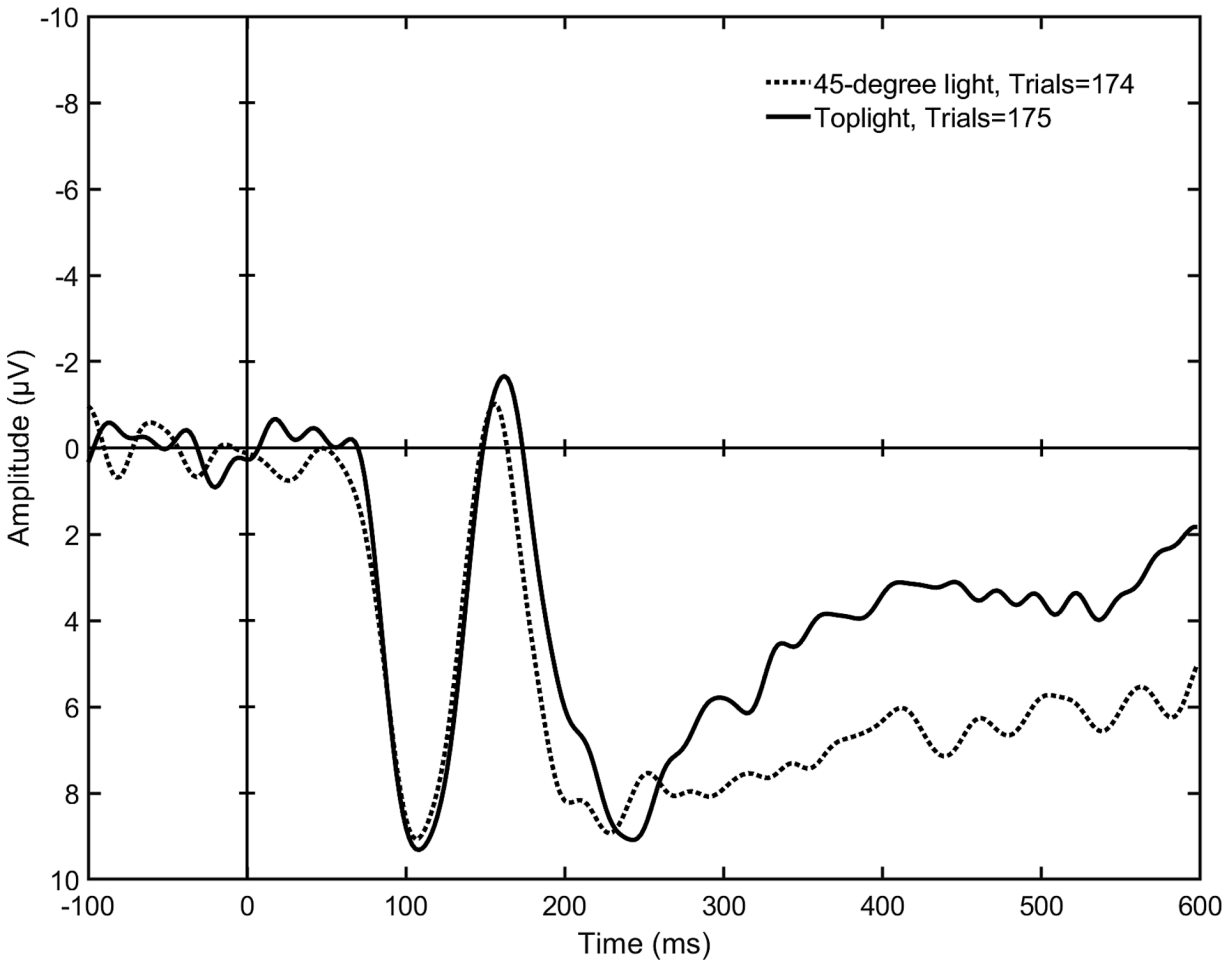
The mean signal amplitudes elicited by toplight (setup 5) and 45-degree light (setup 2) (*N* = 9). The graph shows a visible difference in the mean negativity, especially around 300 ms onwards, indicating that a lighting that comes from above the subject leaving the eyes invisible elicits a stronger early emotional reaction than a lighting that reveals the whole face.

After the statistical tests of the 150–300-ms time span, the ERP graphs were visually evaluated, which revealed visible differences also later in the mean negativity, approximately within 200–600 ms post stimulus (see Figures 6–8). Once again, the Friedman test was first applied to the signal means of this later time span, which showed a difference between all nine lighting styles [*χ*^2^(2) = 15.704, *p* = 0.047]. Then, according to the Wilcoxon test, toplight (setup 5) and silhouette light (setup 8) differed statistically significantly from 45-degree light (*Z* = −2.073, *p* = 0.038 and *Z* = −2.310, *p* = 0.021) indicating, again, that these lighting styles elicit a stronger emotional response than a more typical facial lighting. Also, the difference between underlight (setup 4) and 45-degree light came very close to reaching statistical significance (*Z* = −1.955, *p* = 0.051) suggesting that the elicited negativity of underlight might continue also during this later time span of the EPN (see Figure 7). The test statistics of all pairwise comparisons of the 200–600-ms amplitude means are presented in Table 2.

**Table 2:** Statistical comparisons of the signal means of 200–600 ms after stimulus using the Wilcoxon test; other lighting setups against 45-degree light.

Test statistics for 200–600 ms^a^								
	Frontal light vs 45° light	90° light vs 45° light	Underlight vs 45° light	Toplight vs 45° light	Backlight vs 45° light	Backlight + eyelight vs 45° light	Silhouette vs 45° light	Silhouette + eyelight vs 45° light
*Z*	−1.125^b^	−0.652^b^	−1.955^b^	−2.073^b^	−0.889^b^	−1.125^b^	−2.310^b^	−1.481^b^
Asymp. sig. (2-tailed)	0.260	0.515	0.051	0.038	0.374	0.260	0.021	0.139

a Wilcoxon signed ranks test.

b Based on positive ranks.

The *Z* scores are presented on the first row and the two-tailed asymptotic significances (*p*-values) on the second row. The statistically significant differences in the EPN of toplight (setup 5) and silhouette light (setup 8) compared to 45-degree light (setup 2) are highlighted in yellow and suggest that these lighting styles elicit a stronger emotional response than a more typical facial lighting—together with underlight (setup 4), which came very close to reaching statistical significance (*p* = 0.051) also during this later time span of the EPN.

All lighting setups where the EPN was statistically significantly more pronounced in comparison to 45-degree light are presented as graphs in Figures 6–8. Figure 6 shows a stronger negativity of silhouette light (setup 8) throughout the whole 150 to 600 ms time span suggesting that leaving the face completely dark elicits a stronger subliminal emotional reaction than a lighting that reveals the whole face. The graph in Figure 7 indicates that underlight, a light coming from below the face (setup 4) and altering the facial features from what we are used to seeing under a more typical lighting direction, can also result in a subliminal emotional reaction. The same can be seen in Figure 8, where toplight (setup 5), a light that hides the subject’s eyes, elicits a more pronounced negativity than 45-degree light that reveals them, although somewhat later in the EPN.

The increased negativity elicited by underlight (setup 4), toplight (setup 5), and silhouette light (setup 8) is also present in the voltage maps of Figures 4, 5, where the mean activation of the occipito-parietal areas of the scalp reaches less positive levels with these lighting setups compared to 45-degree light (setup 2). The visible frontal positivity, especially in the case of silhouette light within 200–600 ms (Figure 5) is a result of ocular artifacts, which were not separately cleaned from the data since they mainly affect the frontal channels that were outside the ROI of the study and not used in any analyses.

## 4 Discussion and conclusions

Academics and filmmakers share a common understanding that light is a storytelling tool just as any other element in the film (Bordwell and Thompson, 2008; Monaco, 2009; Landau, 2014; Zettl, 2016; Brown, 2021, 2023). Among other things, these researchers and practitioners point out that with light the filmmaker can emphasize certain story elements, reveal and hide things at particular moments during a scene, make characters, objects and places look appealing or appalling, and with all these aspects help shift the viewer’s feelings to the desired direction.

It is noteworthy that academic sources tend not to turn to experimental, psycho-physiological means for finding answers to the question “Why?”. Instead, they merely tend to state the (presumed) effects of film lighting as common-knowledge facts proven by practice and filmmakers’ intuition and know-how. Lotman (2021) has called this practice-based understanding “experimental heuristics” as it is not based on any academic research or theory but rather on tacit knowledge that has emerged and been adopted and adapted along iterative work in film productions. The apparent lack of academic explanatory interest in film lighting is also evident in Nevill’s (2018, 2021) argument that writing about lighting in moving image production has been unsystematic, under-theorized, and anecdotal and that the academic study of cinematography and film lighting has so far taken only phenomenological, historical, and ethnographic perspectives. These perspectives are of course valuable for the academic research of film lighting, but they lack the explanatory power the more experimental approaches can offer.

Despite these shortcomings, some studies with an experimental perspective on cinematography (Heimann et al., 2014, 2019; Yilmaz et al., 2023) and film lighting (Lotman, 2016; Voodla et al., 2020; Huttunen, 2022) have been reported more recently, and this pilot study aims at adding new results and insight into this procession of studies. In this study the EEG of test subjects was measured while they were exposed to nine facial images lit from different directions using only one main light source to shape the face. Due to the main interest in the possible effects of the direction of the light, only one light source was used to keep the independent variables to their minimum. Although more studies with a larger number of test subjects are needed, the results of this study indicate that lighting styles that hide the eyes or distort facial information from what we are used to seeing in daylight can in themselves elicit a subliminal emotional response in the viewer.

What is also noteworthy is that the underexposed setups 6 and 7 that merely obscured facial features, did not seem to have this effect, as their mean EPN amplitudes did not differ statistically significantly from that of the more typical 45-degree light of setup 2. Worth noticing is also that in the case of a silhouette face, eyelight, the existence of two small dots in place of eyes, seems to be enough to reduce the subliminal emotional response in the viewer, since the silhouette face (setup 8) did elicit a statistically more pronounced EPN than 45-degree light (setup 2), whereas the silhouette face with eyelight (setup 9) did not (see Tables 1, 2 for details).

In accordance with the initial hypotheses, the results of this study indicate that lighting directions and conditions that hide or distort facial information of film characters may help increase the negative feelings linked to these characters already at the subliminal stage of emotional processing. These findings are also in line with the results of the previous study of conscious emotional reactions to facial lighting (Huttunen, 2022), which suggests that some of these subliminal emotional responses may help give rise to consciously felt feelings. It is also possible that this process may further affect the overall experienced mood of a film scene as proposed by Grodal (2007).

Nevertheless, it should be noted that since the experimental stimulus consisted only of photographs of a human face, the study lacked diegetic context (Calbi et al., 2017; McCrackin and Itier, 2018), movement, sound, and preceding and succeeding images, aspects that all contribute to the viewer’s reactions when she is watching a narrative film. Context plays also a role at the subliminal level of emotional processing, since studies indicate that affective context information can influence early ERP’s (Righart and de Gelder, 2008; Diéguez-Risco et al., 2013; Wieser et al., 2010). Future studies may, hence, want to explore the effects of lighting by using film clips or even feature-length films to better generalize the results to a real film-watching setting (see Jääskeläinen et al., 2021; Tikka et al., 2023 for a review). The possible differences between watching faces with a direct versus an averted gaze (Adams and Kleck, 2005; Bublatzky et al., 2017; McCrackin and Itier, 2018, 2021) under different lighting setups should also be studied since averted gaze is more common in narrative fiction films that typically avoid breaking the so-called fourth wall between the viewer and the world depicted in the film (Brown, 2012). Additionally, future studies may want to study other emotional ERP components, such as P100, N170, and LPP (Bublatzky et al., 2017; Schindler and Bublatzky, 2020), as well as assess how the EPN and other early components relate to activity in other brain areas associated with emotional processing such as the amygdala and the prefrontal cortex (Dixon et al., 2017; Šimić et al., 2021). Furthermore, viewers’ subliminal emotional responses to facial lighting could also be studied using other psychophysiological measures such as skin-conductance response (SCR), pupillometry, or facial electromyography (fEMG), all of which have been used before in studying emotional visual stimuli (Cowley et al., 2016).

In conclusion, by examining the effects of light’s directionality on the subliminal emotional responses of individuals – and by focusing especially on the face and how this can provide insights into lighting’s possible influence on psychological processes at the pre-conscious level – this study provides further evidence for the practicing filmmakers’ conviction that light affects the film viewer’s emotions. The findings of this research may also have significant implications beyond film production, particularly in the areas of mental health and psychology.

## Data Availability

The raw data supporting the conclusions of this article will be made available by the authors, without undue reservation.
